# Resveratrol inhibits African swine fever virus replication *via* the Nrf2-mediated reduced glutathione and antioxidative activities

**DOI:** 10.1080/22221751.2025.2469662

**Published:** 2025-02-18

**Authors:** Di Liu, Lian-Feng Li, Huanjie Zhai, Tao Wang, Jing Lan, Mengxiang Cao, Meng Yao, Yijing Wang, Jia Li, Xin Song, Yuan Sun, Hua-Ji Qiu

**Affiliations:** a State Key Laboratory for Animal Disease Control and Prevention, Harbin Veterinary Research Institute, Chinese Academy of Agricultural Sciences, Harbin, People’s Republic of China; b College of Veterinary Medicine, Shanxi Agricultural University, Taigu, People’s Republic of China; c College of Animal Science and Technology, Yangtze University, Jingzhou, People’s Republic of China; d College of Animal Science and Veterinary Medicine, Heilongjiang Bayi Agricultural University, Daqing, People’s Republic of China

**Keywords:** African swine fever virus, oxidative stress, Nrf2 signaling pathway, resveratrol, antiviral activity

## Abstract

African swine fever (ASF) is a highly contagious and severe infectious disease caused by African swine fever virus (ASFV). The disease significantly threatens the sustainable development of the global pig industry. Unfortunately, to date, no safe and efficacious vaccines are commercially available except in Vietnam. Antioxidative stress is a critical factor in antiviral strategies. In this study, we show that ASFV infection elevates the level of reactive oxygen species (ROS) and suppresses the nuclear factor erythroid 2-related factor 2 (Nrf2) signaling pathway *in vitro* and *in vivo*. Moreover, overexpressing Nrf2 can significantly inhibit ASFV replication. Through high-throughput screening of natural small molecules against ASFV, we identify resveratrol (RES), an Nrf2 activator, as a compound capable of inducing the cellular antiviral responses and effectively inhibiting ASFV replication in primary porcine alveolar macrophages (PAMs). Notably, untargeted metabolomics profiling reveals that glutathione emerges as a primary differential metabolite related to the antiviral activities of RES against ASFV. Mechanistically, RES exerts its antiviral effects and attenuates the elevated level of ROS caused by ASFV infection by inducing the production of reduced glutathione (GSH) *via* the activation of the Nrf2 signaling pathway. In conclusion, RES exhibits broad efficacy as a potentially effective compound for inhibiting ASFV infection and alleviating the oxidative stress induced by ASFV infection *via* the Nrf2 signaling pathway.

## Introduction

African swine fever (ASF) is an acute, severe, and highly contagious disease that poses a significant threat to domestic pigs and wild boars of varying ages and breeds. The disease is caused by African swine fever virus (ASFV), which is the sole member of the *Asfarviridae* family and the only known DNA arbovirus [1]. ASFV is a large double-stranded DNA virus with a genome length ranging from 170 to 194 kilobase pairs (kb) [2–5]. The mortality of pigs following infection with ASFV can reach up to 100% [6], causing substantial economic losses to the pig industry and seriously threatening animal food safety. ASF is a notifiable animal disease by the World Organisation for Animal Health (WOAH). The disease has been endemic in Africa and Europe for over a century [7]. It spread to China in 2018, officially marking the introduction of ASFV into the country, and then spread rapidly to neighboring countries due to the lack of effective control measures [8–10].

The severity of ASF highlights the urgent need to identify novel antiviral strategies targeting specific cytokines or signaling pathways. A crucial consideration in antiviral therapies is antioxidative stress, and a growing number of studies have shown that oxidative stress is related to viral infections [11,12]. Most viruses induce elevated levels of reactive oxygen species (ROS) in the infected cells [13–15]. Similarly, ASFV alters the redox homeostasis within infected cells [16]. Oxidative stress can cause cellular damage, resulting in immunopathological changes and exacerbating disease severity [17,18]. Therefore, antiviral strategies should not only aim to inhibit viral replication but also focus on alleviating oxidative stress induced by viral infections.

The nuclear factor erythroid 2-related factor 2 (Nrf2), a transcription factor, is a pivotal regulator in maintaining cellular redox balance by inducing the expression of endogenous antioxidative enzymes in response to oxidative stress [19,20]. Under normal physiological conditions, Nrf2 remains inactive and binds to the Kelch-like epichlorohydrin-associated protein 1 (Keap1) for proteasomal degradation, maintaining low cytosolic levels [21]. However, under oxidative stress conditions, the intracellular ROS level increase, triggering conformational changes in Keap1 that disrupt its binding to Nrf2 [22]. Subsequently, Nrf2 enters the nucleus and binds to the antioxidant response element (ARE) to regulate the expression of antioxidative genes [23]. Typically, the genes regulated by Nrf2 can reduce oxidative stress-induced cell death, rendering Nrf2 a major regulator of tissue damage during viral infections [24]. Notably, it has also been shown that Nrf2 is involved in antiviral responses [25].

Resveratrol (RES), a natural polyphenol derived from plants, has been shown to activate the Nrf2 signaling pathway [26]. It has been demonstrated that the increased cell viability, reduced apoptotic rate, and decreased oxidative stress observed in RES-treated cells were significantly reversed upon Nrf2 knockdown [27]. Besides its antioxidative properties, RES also shows antiviral effects against RNA and DNA viruses, including Zika virus, respiratory syncytial virus, and pseudorabies virus [28–30]. Moreover, it has been demonstrated that RES can protect Vero cells from ASFV infection [31]. However, it remains unclear whether RES inhibits ASFV in target cells and the mechanisms underlying its potential suppression of ASFV replication.

In this study, we demonstrated that ASFV infection promotes the oxidative stress responses, while inhibiting the Nrf2 pathway. However, activation of the Nrf2 pathway can inhibit ASFV replication. By performing the high-throughput screening of natural small molecules against ASFV, we identified RES, an Nrf2 activator, as possessing significant antiviral activity against ASFV. Analysis of untargeted metabolomics data and further experimental validation revealed that RES exerts its antiviral and antioxidative effects through the Nrf2-mediated production of reduced glutathione (GSH). In conclusion, our findings offer a novel approach to the rapidly applicable strategies for the prevention and control of ASFV.

## Materials and methods

### Live virus manipulation facilities and ethics statements

All the experiments with live ASFV in this study were performed in the animal biosafety level 3 (ABSL-3) facilities at Harbin Veterinary Research Institute (HVRI) of the Chinese Academy of Agricultural Sciences approved by the Ministry of Agriculture and Rural Affairs, China. This study was conducted in compliance with the Animal Welfare Act and Guide for the Care and Use of Laboratory Animals, approved by the Laboratory Animal Welfare Committee of HVRI.

### Cells and virus strains

Primary porcine alveolar macrophages (PAMs) isolated from 21 – to 25-day-old specific-pathogen-free (SPF) pigs by bronchoalveolar lavage and wild boar lung (WSL) cells were cultured in RPMI 1640 medium (catalog no. C11875500BT; Gibco) containing 10% fetal bovine sera (FBS) (catalog no. A5669701; Gibco) at 37°C in 5% CO_2_. The ASFV HLJ/2018 strain (ASFV-WT) (GenBank accession no. MK333180.1), isolated from field samples in China as described previously [9], was used in this study. Additionally, various gene-deleted ASFV mutants, including ASFV-ΔCD2v, ASFV-ΔMGF, and ASFV-ΔMGF/CD2v, were constructed using ASFV-WT by our laboratory for further experimentation [5].

### Natural small molecules and antibodies

A total of 128 natural small molecules were provided by Weikeqi Bioscience (Sichuan, China) for screening purposes. Genistein (catalog no. HY-14596), as an ASFV-specific inhibitor, were purchased from MCE. Rabbit anti-A137R polyclonal antibodies were prepared as described previously [32]. Additionally, rabbit anti-Nrf2 (catalog no. A21176), mouse anti-Glyceraldehyde-3-phosphate dehydrogenase (GAPDH) (catalog no. AC033) monoclonal antibodies were purchased from Abclonal.

### Construction of plasmids

To construct the plasmid expressing the Myc-tagged *Nrf2* gene, the *Nrf2* gene from pigs was cloned into the pCMV-Myc vector to generate the expression plasmid pMyc-Nrf2.

### Transfection of WSL cells

WSL cells were seeded into 12-well cell culture plates and cultured overnight. Then, the cells were transfected with 1 and 2 μg of the plasmid pMyc-Nrf2 using X-tremeGENE HP DNA transfection reagent (catalog no. 06366236001; Roche) according to the manufacturer’s instructions.

### ROS assay

The 2,7-dichlorodihydrofluorescein diacetate (DCFH-DA) probe was diluted with serum-free RPMI 1640 medium according to the manufacturer's instructions (catalog no. S0033S; Beyotime). PAMs (2 × 10^5^ cells/well) were infected with ASFV-WT or co-treated with RES and ASFV-WT. And At 12, 24 and 48 hours postinfection (hpi), the cells were washed twice with phosphate-buffered saline (PBS) and replaced with the diluted DCFH-DA solution for 20 minutes at 37°C in the dark. During this period, the cells and the DCFH-DA solution were gently mixed every 3-5 minutes to ensure complete contact. Subsequently, the cells were washed thrice with serum-free RPMI 1640 medium to thoroughly remove any unbound DCFH-DA. The ROS level in the cells was determined using the DCFH-DA probe(excitation/emission = 488/525 nm) and a microplate reader (PE, USA), ensuring that each sample for ROS measurement contained the same number of cells.

### Reverse transcription-quantitative PCR (RT-qPCR)

Total RNAs were extracted from the PAMs infected with ASFV-WT alone or co-treated with RES using an RNA isolation kit (catalog no. BSC52M1; BioFlux), according to the manufacturer’s instructions. For RT-qPCR, cDNA was generated using FastKing gDNA Dispelling RT SuperMix (catalog no. A0530A; Tiangen) and subsequently quantified using the ChamQ SYBR qPCR Master Mix (catalog no. Q311-02; Vazyme). The relative transcriptional levels of each gene were determined using the comparative cycle threshold (2^-ΔΔCT^) method [5]. GAPDH was used as an internal reference control. The primers used for RT-qPCR are listed in Table S1.

### RNA interference assay

Three small interfering RNAs (siRNAs) targeting the porcine *Nrf2* gene were designed and synthesized by Seven Bioscience (Harbin, China) (Table S2). For siRNA transfection, PAMs were transfected with 100 pmol of the siRNAs using X-tremeGENE siRNA transfection reagent (catalog no. 4476115001-1; Roche) according to the manufacturer’s instructions.

### Measurement of intracellular GSH level

The intracellular GSH was determined employing the GSH and oxidized glutathione (GSSG) assay kit (catalog no. S0053; Beyotime), according to the manufacturer’s instructions. Briefly, the PAMs infected with ASFV alone or co-treated with RES were harvested and washed twice with 200 μL of sterilized ice-cold PBS. Subsequently, the cells were mixed with 5% metaphosphoric acid and subjected to two rapid freeze–thaw cycles. The samples were then centrifuged at 10,000 ×*g* for 10 minutes. The supernatants were collected to determine GSH by measuring the optical density at 412 nm (OD_412nm_) using a microplate reader.

### qPCR

The ASFV genomic DNA was extracted from the PAMs infected with ASFV and co-treated with or without RES using a TIANamp genomic DNA kit (catalog no. A0909A; Tiangen), according the manufacturer’s protocols. The ASFV genome was then quantitated by qPCR using the QuantStudio system (Applied Biosystems, USA) based on the WOAH-recommended primers [33]. The viral genome copies in the samples were quantified by targeting the ASFV *B646L* gene and were calculated using a standard curve.

### Generation and identification of the reporter ASFV

The recombinant transfer vector pOK12-p72-CD2v-mRuby was constructed as described previously [34]. Briefly, the left and right flanking fragments were amplified by PCR and assembled to contain the *CD2v* fused with the red fluorescent protein mRuby by overlapping PCR. The expression cassette was subsequently cloned into the linearized pOK12 vector, generating the recombinant transfer vector pOK12-p72-CD2v-mRuby using the ClonExpress II one step cloning kit (catalog no. C112; Vazyme).

The recombinant rASFV-mRuby, serving as a reporter ASFV, was successfully generated by homologous recombination between the genome of ASFV-WT and the recombinant transfer vector by transfection and infection procedures in PAMs [35]. PAMs were cultured in 6-well cell culture plates and transfected with 3 μg of the transfer vector pOK12-p72-CD2v-mRuby using the X-tremeGENE HP DNA transfection reagent. At 24 hours posttransfection (hpt), PAMs were infected with ASFV-WT at a multiplicity of infection (MOI) of 1 for 48 hours. The rASFV-mRuby was purified by successive limiting dilution, and then amplified in PAMs to produce a virus stock. To ensure the fusion expression gene between *CD2v* and *mRuby*, and confirm the absence of any genes deletions in the recombinant genome, viral DNA was extracted from the rASFV-mRuby-infected PAMs and analyzed using PCR with the specific primers targeting these genes, followed by the next-generation sequencing (NGS) analysis to ensure the integrity and accuracy of the genetic modifications.

### Determination of rASFV-mRuby replication kinetics

Comparative growth curve assays between rASFV-mRuby and ASFV-WT were performed in PAMs. Briefly, PAMs were cultured in 24-well cell culture plates and incubated overnight. The cells were then infected with rASFV-mRuby or ASFV-WT at an MOI of 0.01, and washed twice with PBS and replaced with fresh medium at 2 hpi. The infected PAMs were further incubated for various durations (2, 12, 24, 36, 48, 60, 72, 96, 120, or 168 hpi) at 37°C in 5% CO_2_. At the specified time points, both the supernatants and the cell lysates were collected and stored at −80°C. The viral genome copies and titers of ASFV-WT and rASFV-mRuby were quantified by qPCR and hemadsorption (HAD) assays.

### Collection and detection of the ASFV-infected pig tissues

Representative tissues and organs, including heart, liver, spleen, lung, kidney, submandibular lymph nodes (SLN), and mesenteric lymph nodes (MLN), were collected in our previous study [35]. The levels of *Nrf2* and its downstream genes in these tissues were quantitated by RT-qPCR as above.

### Cytotoxicity assay

The cytotoxicity of dimethyl sulfoxide (DMSO), RES, GSH, and L-buthionine-(S,R)-sulfoximine (BSO) to PAMs was evaluated using the CellTiter-Glo (CTG) assay (catalog no. G7572; Promega). PAMs were cultured in 96-well cell culture plates (with a seeding density of 10^5^ cells/well) with varying concentrations of DMSO (0.02%, 0.05%, 0.1%, 0.2%, 0.5%, and 1% of original concentration), RES (10, 20, 40, 80, and 200 μM), GSH (1, 2, 4, and 8 μM), and BSO (2, 5, 10, 20, 50, and 100 μM) for 24 hours at 37°C in 5% CO_2_. After incubation, the medium was discarded, and the cells were washed with sterile PBS. Then, 100 μL of the CTG reagent was added to each well. The cells were incubated at room temperature for 10 minutes, and then 100 μL of the reaction solution was added to each well of black-bottom 96-well cell culture plates (catalog no. BS-MP-96B-CL, Biosharp) to measure the luminescence values using a microplate reader. The cell survival rate was calculated for each concentration according to the following formula: [(treated group – blank group) / (negative control group – blank group)] × 100%.

### Viral titration

PAMs (10^5^ cells/well) were cultured in 96-well cell culture plates and incubated with ASFV of 10-fold serially diluted with the RPMI 1640 medium containing 10% FBS in an incubator containing 5% CO_2_ at 37°C for 5 days. Subsequently, porcine red blood cells (10^6^ cells/well) were added to each well, and the “rosettes” of red blood cells were observed using an optical microscope. The 50% hemadsorption dose (HAD_50_) of ASFV was calculated to evaluate the median tissue culture infectivity of ASFV [36].

### Untargeted metabolomics profiling and liquid chromatography-mass spectrometry (LC-MS/MS) analysis

To analyze the key differential metabolites responsible for inhibiting ASFV replication through RES, PAMs were cultured in 24-well cell culture plates (10^6^ cells/well) and then either were left untreated, infected with ASFV (MOI = 1.5) alone, or co-treated with ASFV (MOI = 1.5) and 40 μM RES. At 24 hpi, the treated PAMs were washed thrice with ice-cold PBS and collected individually in tubes kept on ice. All the samples were centrifuged at 500 ×*g* for 5 minutes at 4°C, and the resulting cell lysates fixed with 4% paraformaldehyde were used for the untargeted metabolomics profiling and LC-MS/MS analysis by Maiteville Bioscience (Wuhan, China). Differential metabolites were determined by variable importance in the projection (VIP) (VIP > 1) and *P* values (*P* < 0.05). VIP values were extracted from the OPLS-DA results, and were generated using the R package MetaboAnalyst. The data was log transform (log_2_) and mean centering before OPLS-DA. In order to avoid over-fitting, a permutation test was performed.

### Western blotting

The PAMs cultured in 12-well cell culture plates were harvested and lysed in ice-cold NP-40 lysis buffer (catalog no. P0013F; Beyotime) supplemented with a protease inhibitor PMSF (1 mM) (catalog no. BL507A; Beyotime) at 4°C for 30 minutes, and centrifuged at 12,000 ×*g* for 15 minutes at 4°C. The supernatants in the loading buffer were boiled for 10 minutes and resolved by sodium dodecyl sulfate-polyacrylamide gel electrophoresis (SDS-PAGE). The resulting proteins were transferred onto polyvinylidene fluoride membranes, followed by blocking with 5% skim milk in Tris-buffered saline containing 0.05% tween 20 (TBST) for 1 hour at room temperature. The membranes were then probed with appropriate primary antibodies, followed by incubation with secondary antibodies. Finally, the signals were visualized using an Odyssey imaging system.

### Statistical analysis

All experiments were performed independently in triplicates, and statistical analysis was performed using SPSS 22.0 software. Differences between groups were examined for statistical significance using unpaired *t* test. The *P* < 0.05 was considered to be significant.

## Results

### ASFV infection induces oxidative stress

**Figure 1.**
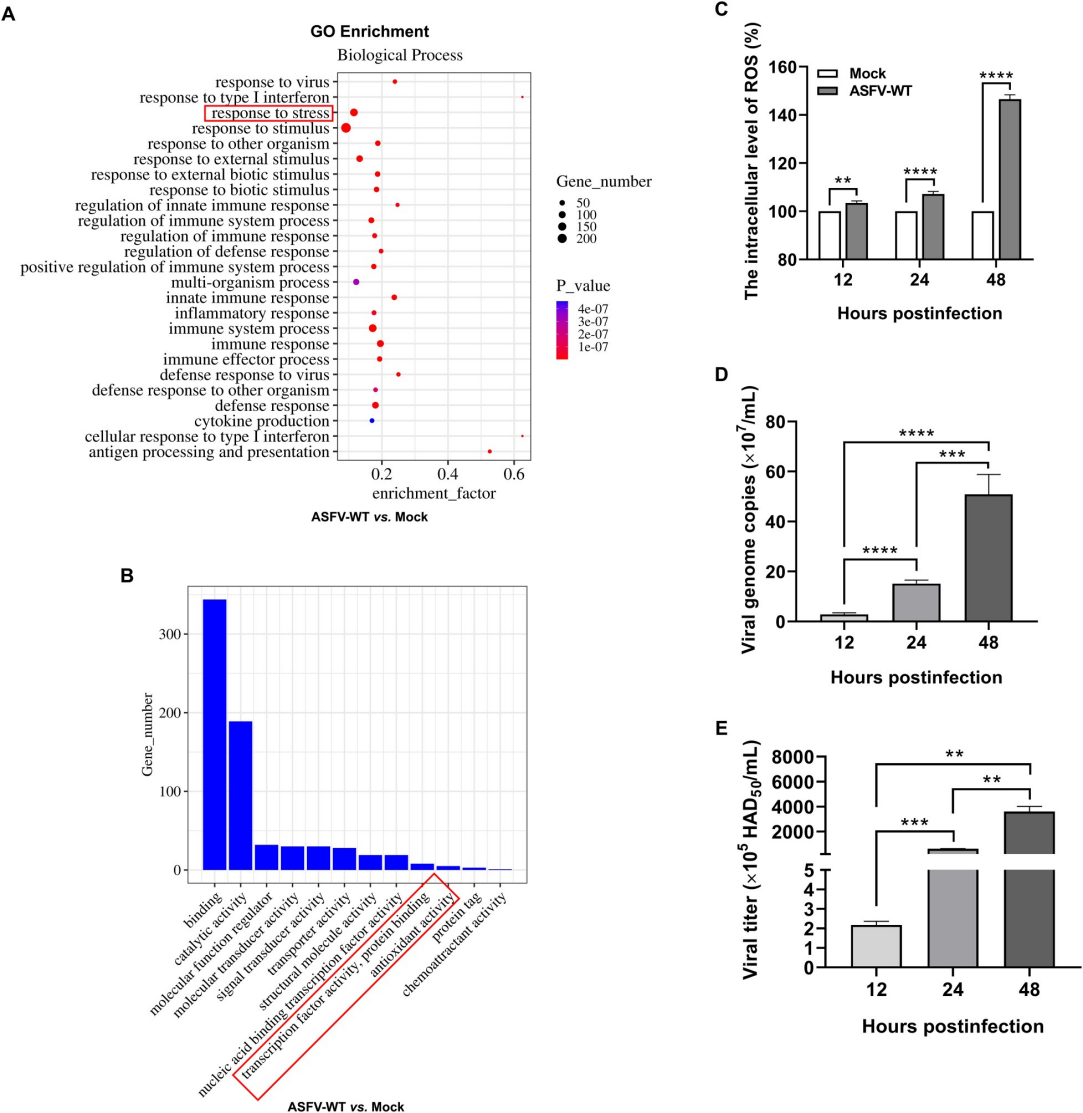
ASFV infection induces oxidative stress. (A and B) Reanalysis of the transcriptome data published by our laboratory. Gene ontology (GO) enrichment analysis was performed between the ASFV HLJ/2018 strain (ASFV-WT)-infected and uninfected primary porcine alveolar macrophages (PAMs) at 12 hours postinfection (hpi). (C) ASFV infection increases the reactive oxygen species (ROS) level. PAMs were infected with ASFV-WT at a multiplicity of infection (MOI) of 1. At 12, 24, and 48 hpi, the intracellular ROS level was measured using the 2,7-dichlorodihydrofluorescein diacetate (DCFH-DA) probe. (D and E) ASFV replication increased with the duration of infection. PAMs were infected with ASFV-WT at an MOI of 1. At 12, 24, and 48 hpi, the viral genome copies (D) and titers (E) were quantified by quantitative PCR and hemadsorption assay, respectively. **, *P* < 0.01; ***, *P* < 0.001; ****, *P* < 0.0001.

To investigate whether ASFV infection induces oxidative stress, we reanalyzed the transcriptome datasets previously made in our laboratory [35]. Compared with the uninfected PAMs, the differentially expressed genes (DEGs) in the PAMs infected with ASFV-WT showed enrichment in cellular response related to the stress signaling cascade (Figure 1(A)) and in the molecular functions related to the transcription factors and antioxidative activities (Figure 1(B)) in the gene ontology (GO) enrichment analysis (red rectangle). Moreover, to determine whether ASFV infection induces the production of ROS, PAMs were infected with ASFV-WT and the ROS level was measured using the DCFH-DA probe at 12, 24, and 48 hpi, respectively. The results demonstrated that the ROS level in the ASFV-infected PAMs was elevated significantly (Figure 1(C)), along with an increase in the viral genome copies (Figure 1(D)) and titers (Figure 1(E)), compared with the control group. Collectively, these data suggest that ASFV infection induces oxidative stress.

### ASFV infection suppresses the Nrf2 signaling pathway

**Figure 2.**
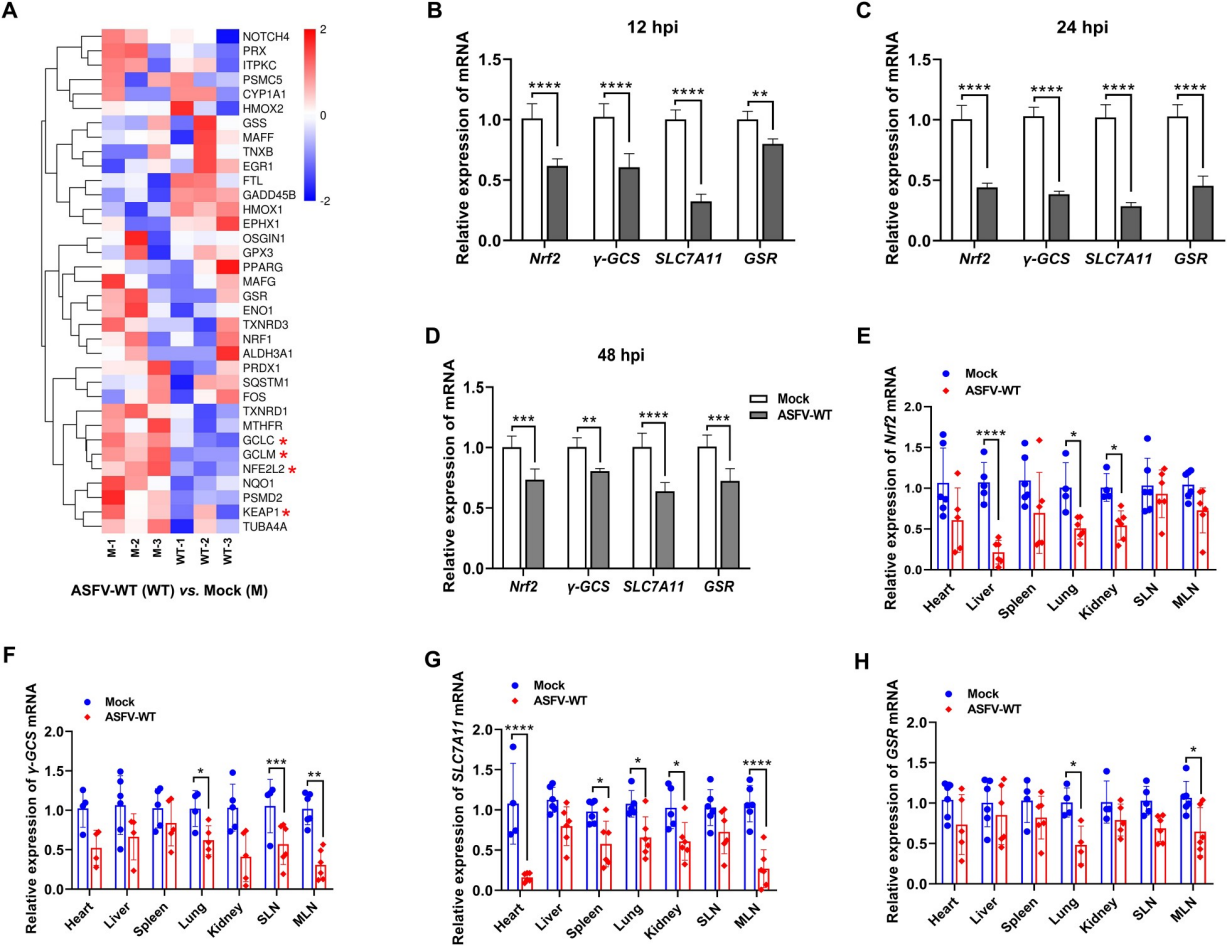
ASFV infection suppresses the Nrf2 signaling pathway. (A) Heatmap of the differentially expressed genes (DEGs) induced by the ASFV HLJ/2018 strain (ASFV-WT) at 12 hours postinfection (hpi). (B–D) ASFV infection suppresses the Nrf2 signaling pathway in primary porcine alveolar macrophages (PAMs). PAMs were infected with ASFV-WT at a multiplicity of infection (MOI) of 1. The cells were collected to quantify the mRNA levels of *Nrf2* and its target genes by reverse transcription-quantitative PCR (RT-qPCR) at 12 (B), 24 (C), and 48 (D) hpi. (E–H) ASFV infection inhibits the Nrf2 signaling pathway in pigs. The mRNA levels of *Nrf2* (E), *γ-GCS* (F), *SLC7A11* (G), and *GSR* (H) in each pig uninfected and infected with ASFV-WT were quantified by RT-qPCR. MLN, mesenteric lymph node; SLN, submandibular lymph node. *, *P* < 0.05; **, *P* < 0.01; ***, *P* < 0.001; ****, *P* < 0.0001.

Considering that the Nrf2 pathway is a major cellular defense system against oxidative stress [37], the heatmap of DEGs was reanalyzed based on the aforementioned transcriptome datasets. The results demonstrated that ASFV-WT infection suppresses key genes involved in the Nrf2 signaling pathway, including *GCLM*, *GCLC*, *Keap1*, and *NFE2L2* (*Nrf2*) (Figure 2(A)). To determine whether ASFV infection inhibits the Nrf2 signaling pathway both *in vitro* and *in vivo*, PAMs were infected with ASFV-WT to confirm the transcriptional levels of *Nrf2* and its downstream genes. Compared with the uninfected PAMs, the mRNA levels of the *Nrf2*, *γ-GCS*, *SLC7A11*, and *GSR* genes in the PAMs infected with ASFV-WT were repressed at 12, 24, and 48 hpi (Figure 2(B–D)). Furthermore, we ground tissues from the pigs infected with ASFV-WT previously conducted by our laboratory for RT-qPCR [35]. The results showed that the transcriptional levels of the *Nrf2*, *γ-GCS*, *SLC7A11*, and *GSR* genes were decreased in the pigs infected with ASFV-WT, compared with the uninfected group (Figure 2(E–H)). Altogether, these data indicate that the Nrf2 signaling pathway is inhibited during the ASFV infection.

### Nrf2 inhibits the replication of ASFV in WSL cells

**Figure 3.**
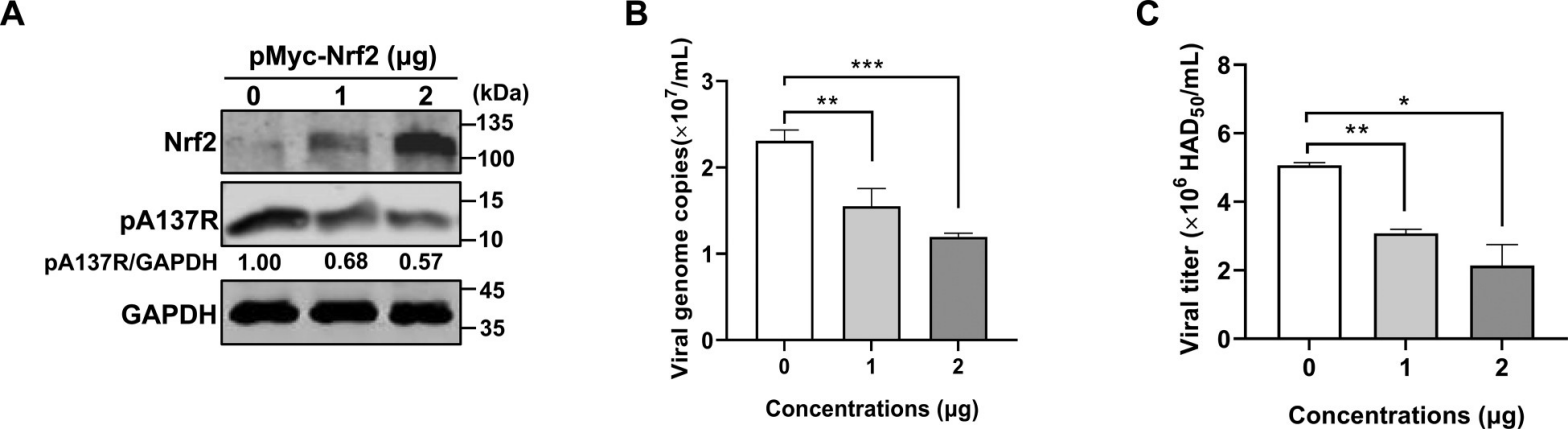
Nrf2 inhibits ASFV replication. Wild boar lung (WSL) cells were transfected with pMyc-Nrf2 or pCMV-Myc. At 24 hours posttransfection, the WSL cells were infected with the ASFV HLJ/2018 strain (ASFV-WT) at a multiplicity of infection (MOI) of 1. At 24 hours postinfection, the cells and the supernatants were collected to determine the expression of the ASFV A137R protein (A), the viral genome copies (B), and viral titers (C) by Western blotting, quantitative PCR (qPCR), and hemadsorption (HAD) assay, respectively. *, *P* < 0.05; **, *P* < 0.01; ***, *P* < 0.001.

Given that the Nrf2 signaling pathway is suppressed during ASFV infection, we sought to investigate whether Nrf2 inhibits ASFV replication. WSL cells, which show a higher transfection efficiency compared with PAMs, were transfected with pMyc-Nrf2 of different amounts. At 24 hpt, the cells were infected with ASFV-WT at an MOI of 1 to perform Western blotting, qPCR, and HAD assays at 24 hpi. The results revealed that overexpression of Nrf2 significantly reduced the ASFV A137R expression (Figure 3(A)), the viral genome copies (Figure 3(B)), and titers (Figure 3(C)), suggesting that Nrf2 significantly inhibits the replication of ASFV in WSL cells.

### Screening of Nrf2 activators against ASFV

Since compounds discovered from phenotypic assays at the cellular level are more likely to be efficacious than target-based approaches, we developed a high-throughput screening platform based on a phenotypic approach for anti-ASFV, with the goal of identifying compounds that can activate Nrf2. Initially, we constructed a fluorescent reporter rASFV-mRuby and confirmed its purification through fluorescence observation (Figure S1A) and PCR identification (Figure S1B). The replication kinetics of rASFV-mRuby was comparable to those of ASFV-WT (Figure S1C and D). Additionally, the NGS analysis revealed that there were no undesired mutations apart from the insertion of the fused *mRuby* gene in the rASFV-mRuby genome (Figure S1E). Subsequently, we used rASFV-mRuby to establish a high-throughput screening platform for screening small molecules against ASFV.

Given that the 128 small molecules being evaluated were dissolved in DMSO, we determined the noncytotoxic concentration of DMSO to PAMs using the CTG assay. The results showed that DMSO concentration of no more than 0.2% did not induce any significant cytotoxicity (Figure S1F). Meanwhile, we employed the Z factor to identify the optimal infective dose of rASFV-mRuby for the high-throughput screening platform. The results indicated that PAMs incubated with rASFV-mRuby at an MOI of 1.5 for 24, 36, or 48 hours yielded a Z factor value ranging from 0.5–1, which were deemed suitable for the assay (Figure S1G). Additionally, the greatest difference between the negative and positive controls was observed for 24 hours, highlighting a significant contrast in ASFV infection fluorescence (Figure S1H). Thus, the condition of high-throughput screening platform that the PAMs incubated with rASFV-mRuby at an MOI of 1.5 for 24 hours meets the screening criteria for the compounds.

Afterward, 128 compounds were screened using the platform based on the cell imaging system (Figure S2A). Among them, 7 compounds exhibited notable inhibitory effects on ASFV, with RES being the only Nrf2 activator (Figure S2B). The chemical structure of RES is shown in Figure S2C. Furthermore, we determined the effect of RES on the viability of PAMs at various concentrations and calculated the 50% cytotoxic concentration (CC_50_). The results showed that concentrations of RES up to 40 μM had minimal impact on the viability of PAMs (Figure S2D), with the CC_50_ of 143.1 μM (Figure S2E).

### RES inhibits ASFV replication

**Figure 4.**
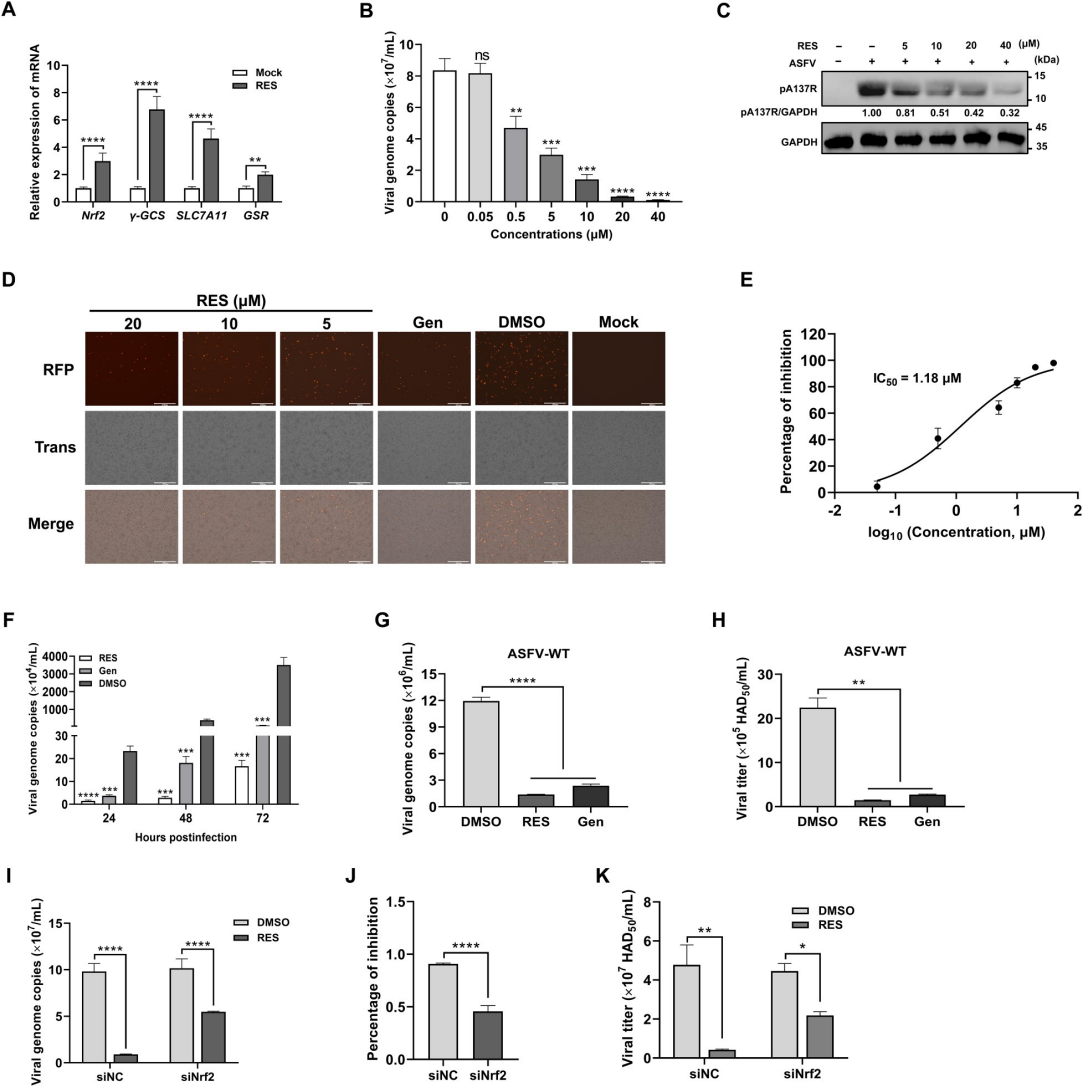
Resveratrol (RES) inhibits ASFV replication. (A) RES activates the Nrf2 signaling pathway. Primary porcine alveolar macrophages (PAMs) were treated with 40 μM RES for 48 hours, and the cells were collected to detect the mRNA levels of Nrf2 and its target genes. (B–E) RES inhibits ASFV replication in a dose-dependent manner. PAMs were infected with the fluorescent reporter ASFV (rASFV-mRuby) at a multiplicity of infection (MOI) of 1.5 together with different concentrations of RES, and incubated for 24 hours to determine the viral genome copies (B), the expression of the ASFV A137R protein (C), fluorescence (D), and the 50% inhibitory concentration (IC_50_) of RES (E). (F) RES inhibits ASFV replication in a time-dependent manner. PAMs were co-treated with rASFV-mRuby (MOI = 0.1) and RES (40 μM) for 24, 48, and 72 hours, and then the supernatants and the cell lysates were collected to quantify viral genome copies by quantitative PCR. (G and H) RES inhibits the replication of the ASFV HLJ/2018 strain (ASFV-WT). PAMs were co-treated with RES (40 μM) and ASFV-WT (MOI = 1.5). At 24 hours postinfection (hpi), the supernatants and the cell lysates were collected to quantify viral genome copies (G) and titers (H). (I-K) The knockdown of Nrf2 impairs the ability of RES inhibiting ASFV replication. PAMs were transfected with siNrf2 or siNC, and then either infected with ASFV-WT (MOI = 1) or co-treated with RES (40 μM) and ASFV-WT (MOI = 1) at 24 hours posttransfection. At 24 hpi, the supernatants and the cell lysates were collected to quantify viral genome copies (I), the inhibitory rates (J), and viral titers (K). *, *P* < 0.05; **, *P* < 0.01; ***, *P* < 0.001; ****, *P* < 0.0001; ns, not significant.

To confirm that RES can indeed activate the Nrf2 signaling pathway, PAMs treated with 40 μM RES for 48 hours were collected to quantify the mRNA levels of *Nrf2* and its target genes, including *γ-GCS*, *SLC7A11*, and *GSR*. The results confirmed that RES effectively activated the Nrf2 signaling pathway (Figure 4(A)). Then, to further assess the antiviral activities of RES against ASFV, we co-introduced rASFV-mRuby (MOI = 1.5) and different concentrations of RES into PAMs for 24 hours. The viral genome copies (Figure 4(B)), the expression of the ASFV A137R protein (Figure 4(C)), and the fluorescence observation (Figure 4(D)) indicated that RES effectively inhibited the replication of ASFV and the 50% inhibitory concentration (IC_50_) of RES was 1.18 μM (Figure 4(E)). In addition, we calculated the selective index (SI, CC_50_/IC_50_) of RES as 121.27, indicating that RES possesses significant potential as a promising anti-ASFV compound. Beyond that, RES significantly inhibited the replication of ASFV at 24, 48, and 72 hpi, exhibiting sustained inhibitory effects (Figure 4(F)). Moreover, it also exhibits antiviral activities against both ASFV-WT (Figure 4(G and H)) and different gene-deleted ASFV strains, including ASFV-ΔMGF (Figure S3A and B), ASFV-ΔCD2v (Figure S3C and D), and ASFV-ΔMGF/CD2v (Figure S3E and F).

To further evaluate whether RES inhibits the replication of ASFV by the Nrf2 signaling pathway, three distinct siRNAs (siNrf2) specifically targeting the *Nrf2* gene were transfected into PAMs to measure which siRNA had the most efficient interference. The results showed that siNrf2-2 exhibited the most substantial knockdown efficiency (Figure S4A), and the transfection of the three siRNAs, at the concentrations tested, did not induce any significant cytotoxicity (Figure S4B). Subsequently, PAMs were transfected with siNrf2-2. At 24 hpt, the PAMs were either infected with ASFV-WT or co-treated with RES and ASFV-WT. The supernatants were collected to quantify the viral genome copies and titers at 24 hpi. The results showed that siRNA silencing of Nrf2 reduced the capacity of RES to inhibit ASFV replication (Figure 4(I–K)), suggesting that Nrf2 contributes to the inhibitory effect of RES against ASFV.

### Glutathione serves as the major differential metabolite of RES inhibiting ASFV replication in untargeted metabolomic analysis

**Figure 5.**
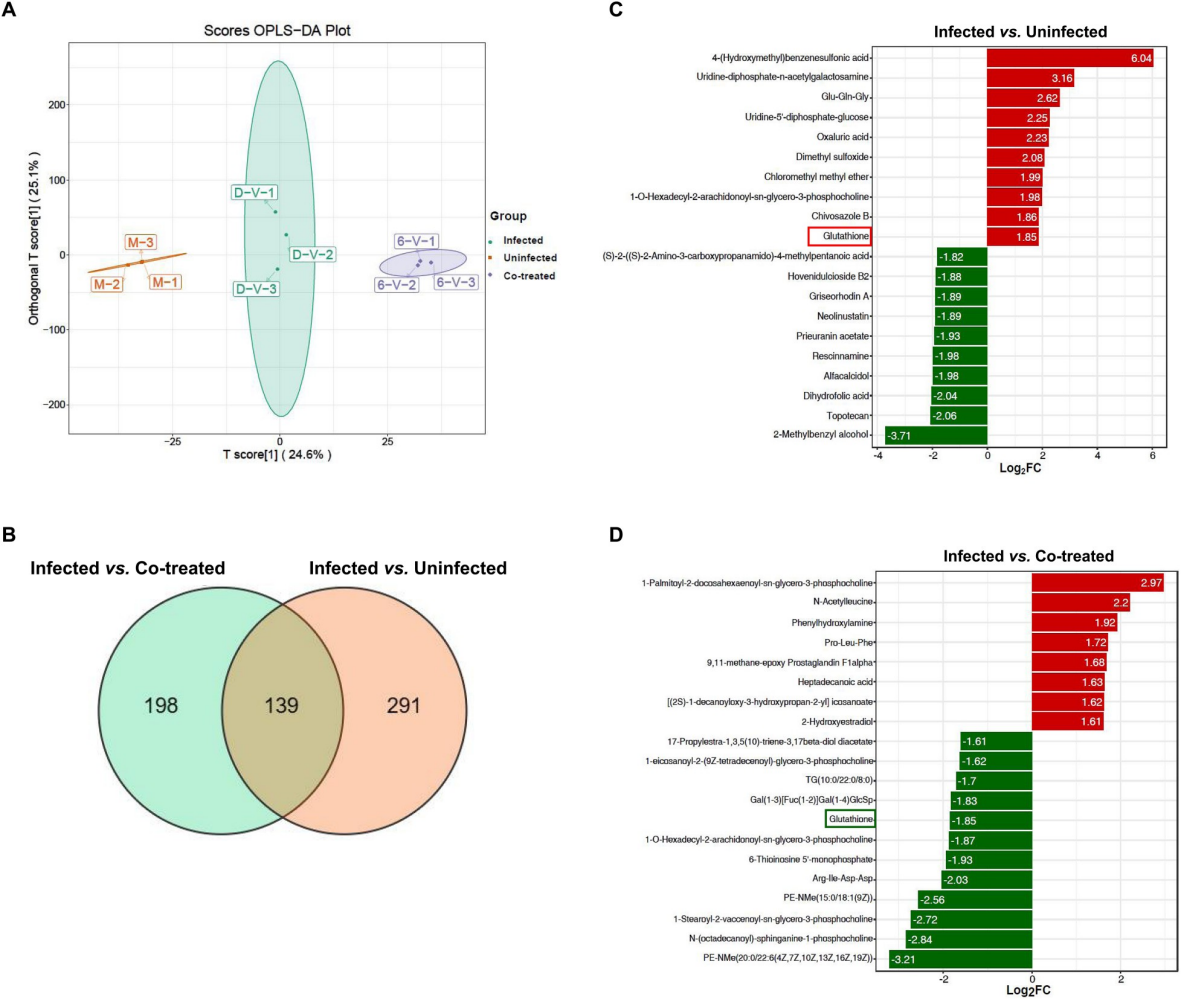
Glutathione is a primary differential metabolite of resveratrol (RES) to inhibit ASFV replication by the untargeted metabolomic analysis. (A) orthogonal partial least squares-discriminant analysis (OPLS-DA) of three groups. (B) The Venn diagram analysis of the metabolites with significantly different levels that are shared or unique among the different comparisons. (C and D) Fold change analysis of among the top 20 differential metabolites under two groups: between the uninfected and ASFV-infected group (C) and between the ASFV-infected and RES and ASFV co-treated group (D). The red bars indicate upregulated metabolites, and the green bars represent downregulated metabolites.

To explore how RES suppresses ASFV replication depending on Nrf2, PAMs were either left uninfected, infected with ASFV-WT (MOI = 1.5) alone, or co-treated with ASFV-WT (MOI = 1.5) and 40 μM RES for 24 hours, followed by an untargeted metabolomics analysis. The orthogonal partial least squares-discriminant analysis (OPLS-DA) demonstrated that all groups were separated from each other and the respective samples clustered together, indicating that ASFV infection or the co-treatment of RES and ASFV altered the cellular metabolism (Figure 5(A)). Venn diagrams analysis, based on significantly differential metabolites in the two comparative groups, revealed metabolites that are either commonly present or unique to each comparison group. Meanwhile, a total of 139 significantly differential metabolites shared among the two comparative groups (Figure 5(B)). Among the top 20 of these 139 differential metabolites, glutathione as the sole common differential metabolite, such as L-glutamic acid, dopamine, and glutathione. Specifically, glutathione was markedly upregulated during ASFV infection (Figure 5(C)), while its level was downregulated in the PAMs co-treated with RES and ASFV compared with ASFV infection state alone (Figure 5(D)). These results demonstrated that glutathione is the main differential metabolite involved in the inhibition of viral replication by RES.

### RES inhibits ASFV replication through the Nrf2-mediated GSH production

**Figure 6.**
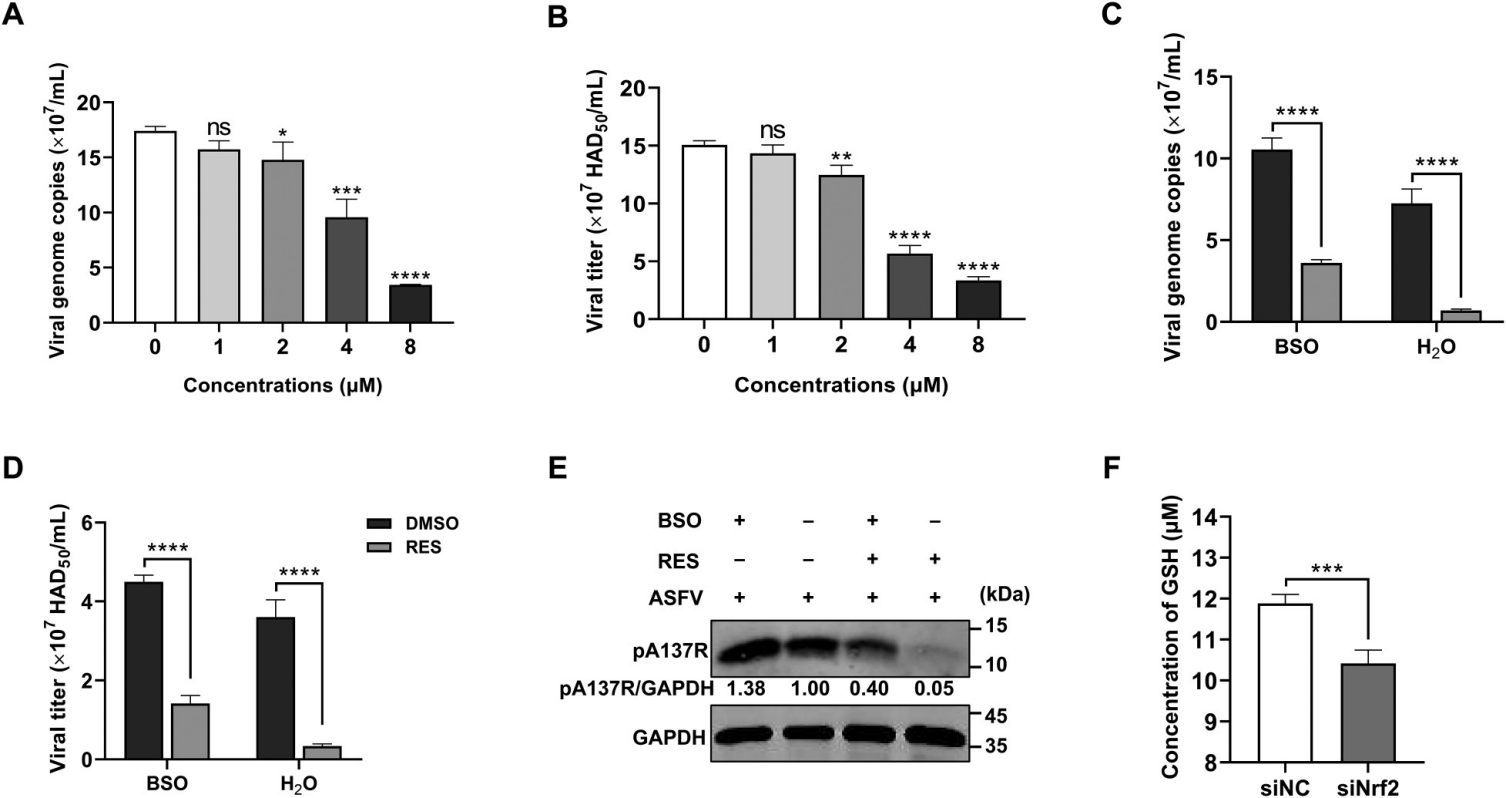
Resveratrol (RES) inhibits ASFV replication by the Nrf2-mediated GSH production. (A and B) GSH inhibits ASFV replication in a dose-dependent manner. Primary porcine alveolar macrophages (PAMs) were co-treated with different concentrations of GSH (1, 2, 4, and 8 μM) and the ASFV HLJ/2018 strain (ASFV-WT) at a multiplicity of infection (MOI) of 1 for 24 hours. The supernatants and the cell lysates were collected for the measurement of the viral genome copies (A) and titers (B). (C–E) The inhibition of GSH synthesis alleviates the ability of RES to suppress ASFV replication. PAMs were treated with GSH synthesis inhibitor BSO (5 μM) for 18 hours. Following that, PAMs were infected with ASFV-WT (MOI = 1) and co-treated with or without RES (40 μM). Then, the supernatants and the cell lysates were collected to determine viral genome copies (C), viral titers (D), and the expression of the ASFV A137R protein (E). (F) The knockdown of Nrf2 decreases the GSH level in PAMs. PAMs were transfected with siNrf2-2 or siNC. At 24 hours posttransfection, the cells were collected to measure the GSH level. *, *P* < 0.05; **, *P* < 0.01; ***, *P* < 0.001; ****, *P* < 0.0001; ns, not significant.

To investigate the role of glutathione, particularly its reduced form GSH as an antioxidant, on ASFV replication, we firstly assessed the safe concentration of GSH using the CTG assay for PAMs. The results showed that GSH has still no cytotoxicity to PAMs at a concentration of 8 µM (Figure S5A). Subsequently, PAMs were treated with different concentrations of GSH (1, 2, 4, and 8 μM) along with ASFV-WT (MOI = 1), and the supernatants and the cell lysates were collected to quantify viral genome copies and titers at 24 hpi. The results demonstrated that GSH inhibited ASFV replication in a dose-dependent manner (Figure 6(A and B)). In contrast, to further evaluate the impacts of inhibiting GSH synthesis on ASFV replication, varying concentrations of GSH synthesis inhibitor BSO were incubated with PAMs for 48 hours to detect its noncyotoxic concentration. No cytotoxicity was observed when the BSO concentration was ≤ 5 μM (Figure. S5B). We then determined the GSH level in the BSO-treated PAMs using the GSH and GSSG assay kit. As expected, the GSH level was markedly downregulated in PAMs (Figure S5C). Following this, PAMs were pretreated with 5 μM BSO and then were infected with ASFV-WT (MOI = 1). At 24 hpi, the effect of BSO on ASFV replication was assessed. The viral genome copies (Figure S5D) and titers (Figure S5E) revealed that BSO facilitated ASFV replication, further suggesting that GSH can inhibit ASFV replication.

To explore whether RES inhibits ASFV replication through the production of GSH, PAMs were treated with BSO (5 μM) for 18 hours to suppress the GSH synthesis, followed by the infection of either ASFV-WT (MOI = 1) or the combination of ASFV-WT (MOI = 1) and RES to the PAMs. Then, the supernatants and the cell lysates were collected for the determination of viral genome copies, viral titers, and the expression of the ASFV A137R protein, respectively. The results showed that BSO treatment reduced the ability of RES to inhibit ASFV replication in PAMs (Figure 6(C–E)). It has been demonstrated that Nrf2 knockdown decreases the antiviral activity of RES against ASFV. To probe whether RES exerts its antiviral effects through Nrf2-mediated GSH production, PAMs were transfected with siNrf2-2 and measured the GSH level using the GSH and GSSG assay kit at 24 hpt. The results showed that knockdown of Nrf2 significantly decreased the intracellular GSH level in PAMs (Figure 6(F)). These results indicated that RES exerts its antiviral activities through Nrf2-mediated GSH production.

### RES reduces the ROS level induced by ASFV infection depending on the Nrf2-mediated GSH production

**Figure 7.**
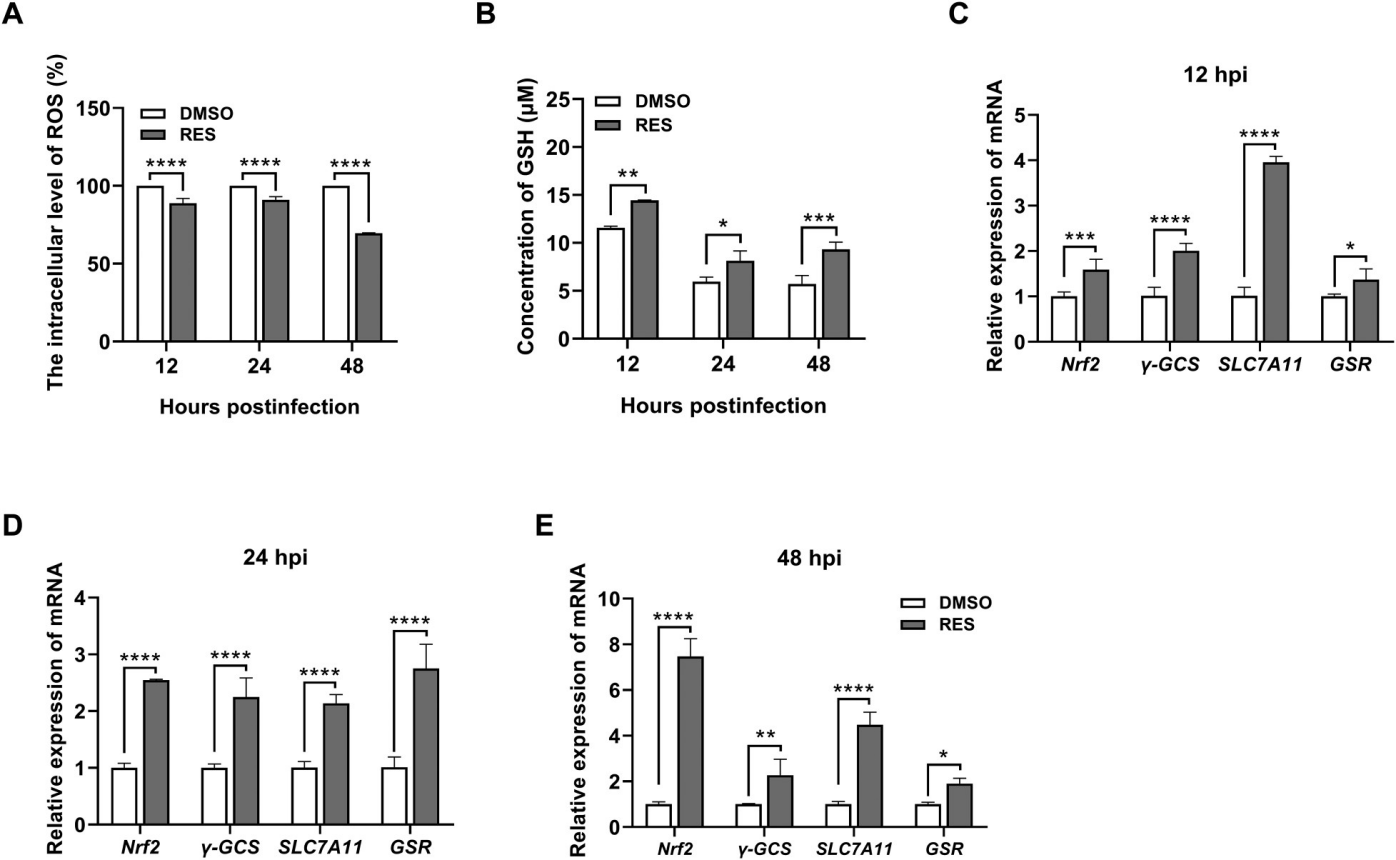
Resveratrol (RES) limits the ROS level induced by ASFV infection through the Nrf2-mediated GSH production. (A and B) RES downregulates the ROS level and upregulates the GSH level during ASFV infection. Primary porcine alveolar macrophages (PAMs) were infected with the ASFV HLJ/2018 strain (ASFV-WT) at a multiplicity of infection (MOI) of 1 or co-treated with RES (40 μM) and ASFV-WT (MOI = 1). At 12, 24, and 48 hours postinfection, the ROS (A) and GSH (B) levels were measured. (C–E) RES significantly increased the mRNA levels of *Nrf2* and its target genes during ASFV infection. PAMs were infected with ASFV-WT at an MOI of 1 or co-treated with RES (40 μM) and ASFV-WT (MOI = 1) for 12 (C), 24 (D), and 48 (E) hours. The cells were collected to quantify the mRNA levels of *Nrf2* and its target genes by reverse transcription-quantitative PCR. *, *P* < 0.05; **, *P* < 0.01; ***, *P* < 0.001; ****, *P* < 0.0001.

To explore whether RES can reduce the ROS level through GSH during ASFV infection, PAMs were infected with ASFV-WT and co-treated with or without RES to perform the measurement of ROS level at specific time points post-infection. The results showed that RES significantly decreased the intracellular ROS level during ASFV infection at 12, 24, and 48 hpi (Figure 7(A)). Meanwhile, during this period, RES also increased the intracellular GSH level in the cells (Figure 7(B)). To further investigate whether the antioxidative effect of RES is mediated through the activation of the Nrf2 signaling pathway, the PAMs infected with ASFV-WT and co-treated with or without RES were collected for RT-qPCR analysis. The results displayed that RES significantly increased the mRNA levels of *Nrf2* and its target genes, including *γ-GCS*, *SLC7A11*, and *GSR*, compared with the ASFV-WT infection group at 12 (Figure 7(C)), 24 (Figure 7(D)), and 48 (Figure 7(E)) hpi. These findings indicated that RES promotes the production of GSH by activating the Nrf2 signaling pathway, thereby exerting the antioxidative effects.

## Discussion

Oxidative stress is a common cellular response to viral infection [38]. Sustained viral infection leads to the accumulation of the ROS in cells or tissues, which can result in tissue damage and cell death [39]. Expanding evidence suggests that excessive ROS production plays a central role in causing local or systemic tissue damage and exacerbates to the severity of viral infections, such as severe acute respiratory syndrome coronavirus 2 (SARS-CoV-2) [40], Epstein – Barr virus [41], and Hepatitis B virus [42]. Similarly, the reanalysis of the transcriptome datasets has confirmed that ASFV infection is associated with oxidative stress responses (Figure 1(A and B)), which may exacerbate the pathogenic consequences of ASF. Additionally, *in vitro* experiment demonstrated that ASFV infection could indeed induce the accumulation of intracellular ROS (Figure 1(C–E)). Given that Nrf2 is an antioxidative transcription factor that regulates endogenous systems responsible for antioxidative defense [43], it is interesting to explore whether the accumulation of ROS induced by ASFV infection is due to a potential dysregulation of the Nrf2 pathway. This could represent a main factor contributing to the oxidative stress during ASFV infection.

It has been reported that activation of the Nrf2/HO-1 pathway can inhibit the replication of porcine reproductive and respiratory syndrome virus [44]. Considering the importance of the Nrf2 transcription factor in antioxidative defense during ASFV infection, we explored whether Nrf2 can inhibit ASFV replication. Through the overexpression of Nrf2, we found that Nrf2 is capable of suppressing ASFV replication (Figure 3). Consequently, we sought Nrf2 activators as potential therapeutic agents against ASFV. In the process of discovering antiviral compounds, phenotypic screening assays are particularly effective in directly and efficiently demonstrating the potential therapeutic efficacy [45]. Therefore, we established a high-throughput screening platform based on a fluorescent reporter ASFV rASFV-mRuby and successfully identified RES as a compound that significantly inhibits ASFV replication by activating the Nrf2 signaling pathway (Figure 4). However, knockdown of Nrf2 did not completely inhibit the anti-ASFV activity of RES (Figure 4(I–K)), suggesting that additional mechanisms may be involved in different stages of viral replication. Notably, RES exhibits a relatively low IC_50_ for inhibiting ASFV replication (Figure 4(E)), and previous studies have reported its strong antioxidative activity [46], highlighting its potential clinical value. This enables RES to be tested as a small-molecule inhibitor of ASFV replication and oxidative stress-induced pathology in animal trials.

Metabolism is important for the efficacy, safety, and pharmacokinetic properties of agents. Metabolic differences among individuals can affect the resistance of agents to diseases, emphasizing the importance of individualized dose [47]. To investigate how RES inhibits ASFV replication through the Nrf2 signaling pathway, we performed LC-MS/MS analysis of untargeted metabolomics and identified glutathione as the key differential metabolite in two major comparisons: between the uninfected and ASFV-infected group, and between the ASFV-infected and RES and ASFV co-treated group (Figure 5(C and D)). Glutathione primarily consists of two forms: GSH and GSSG, with GSH being the predominant form [48]. As a well-known antioxidant, we further identified that GSH inhibits ASFV replication in a dose-dependent manner (Figure 6(A and B)) and the antiviral effects of RES are dependent on GSH (Figure 6(C–E)), which is consistent with several previous reports. For instance, influenza virus, human immunodeficiency virus (HIV), and SARS-CoV-2, impair the metabolism of cellular glutathione, and glutathione can inhibit their replication [49–51]. However, after inhibiting GSH synthesis with BSO, RES did not completely lose the antiviral activities against ASFV, suggesting that in addition to the GSH that was not fully inhibited by BSO, other factors may contribute to the effects of RES to inhibit ASFV replication. For example, it has been reported that RES may inhibit the replication of Middle East respiratory syndrome coronavirus by activating sirtuin proteins [52], while the impact of sirtuin proteins on ASFV replication remains to be explored. Metabolomic analysis identified the different metabolites that could contribute to the antiviral state of RES treated PAMs, which needs the further studies to elucidate the contribution in anti-ASFV. Furthermore, we found that the silencing of Nrf2 reduces the intracellular GSH level (Figure 6(F)), indicating that the antiviral effect of RES against ASFV is at least dependent on Nrf2-mediated GSH production. Meanwhile, we also discovered that RES increases the intracellular GSH level by activating the Nrf2 signaling pathway, thereby exerting the antioxidative effects (Figure 7).

**Figure 8.**
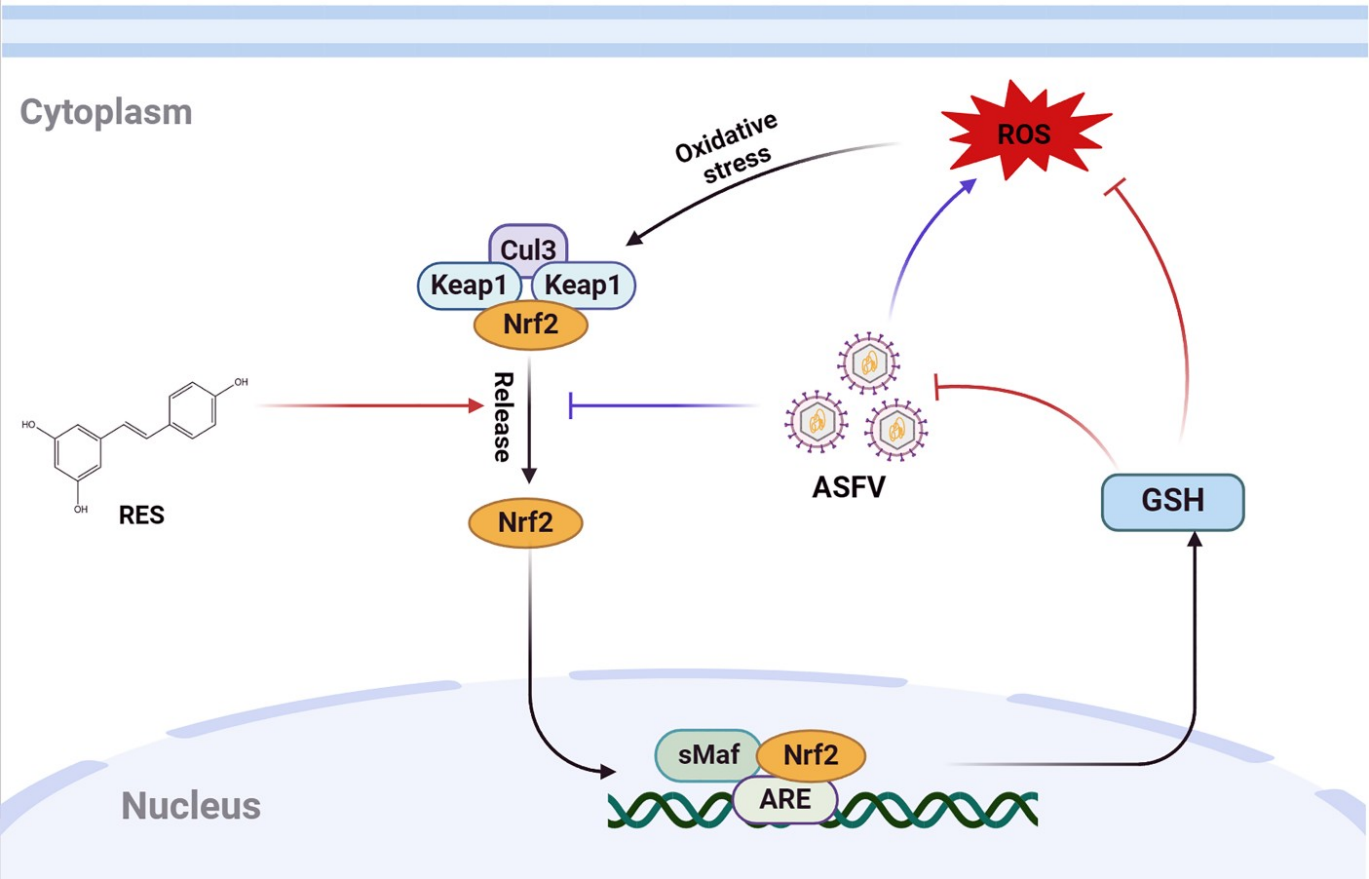
A proposed model of resveratrol (RES) exerts antiviral effects against ASFV and alleviates oxidative stress induced by ASFV infection through the Nrf2 signaling pathway. Black arrows indicate that oxidative stress caused by increased ROS level activates the Nrf2 signaling pathway. ASFV infection suppresses the Nrf2 signaling pathway, resulting in oxidative stress. RES induces the production of GSH by activating the Nrf2 signaling pathway, which in turn inhibits ASFV replication and reduces the ROS level induced by ASFV infection.

In conclusion, we firstly reported and characterized that ASFV infection suppresses the Nrf2 signaling pathway, leading to oxidative stress, which may exacerbate disease progression. As an activator of Nrf2, RES exerts anti-ASFV and antioxidant activities through activation of the Nrf2-GSH signaling axis (Figure 8). The two characteristics may be unique for Nrf2 activators, highlighting their potential to control virus-induced pathology. These findings provide new insights and strategies for the prevention and control of ASF, which could be valuable in alleviating the oxidative stress caused by viral infections.

## Supplementary Material

Figure S2.tif

Figure S1.tif

Figure S3.tif

Table S1.docx

Figure S5.tif

Table S2.docx

Figure S4.tif

## Data Availability

All data needed to evaluate the conclusions in the paper are present in the paper and the Supplementary information.
