# Supplementary figures and images for: Resveratrol inhibits African swine fever virus replication via the Nrf2-mediated reduced glutathione and antioxidative activities
Source: Emerg Microbes Infect. 2025 Feb 18;14(1):2469662. doi: 10.1080/22221751.2025.2469662 (PMC11878180; doi:10.1080/22221751.2025.2469662)

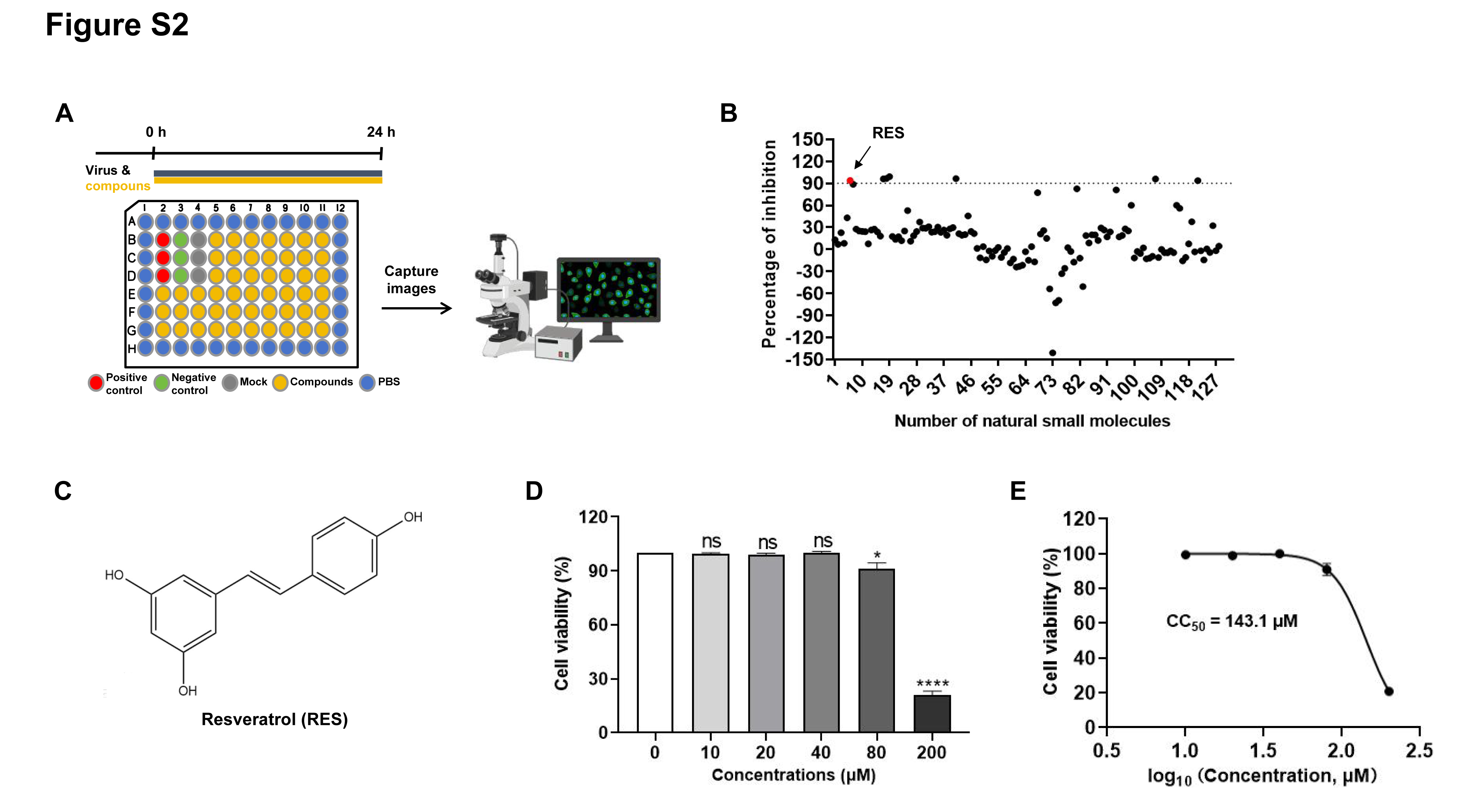

Supplement: Figure S2.tif [file TEMI_A_2469662_SM8778.tif]

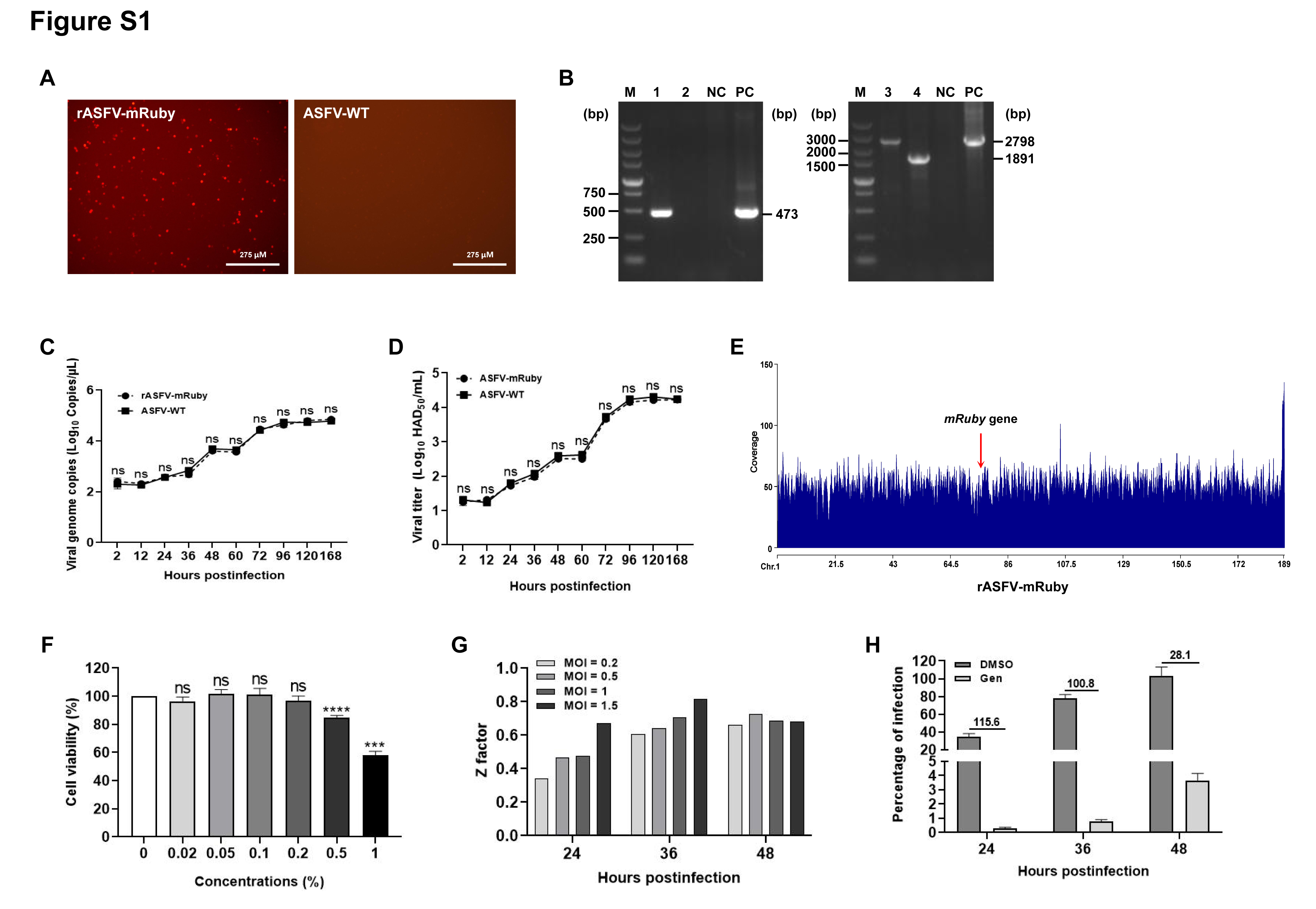

Supplement: Figure S1.tif [file TEMI_A_2469662_SM8777.tif]

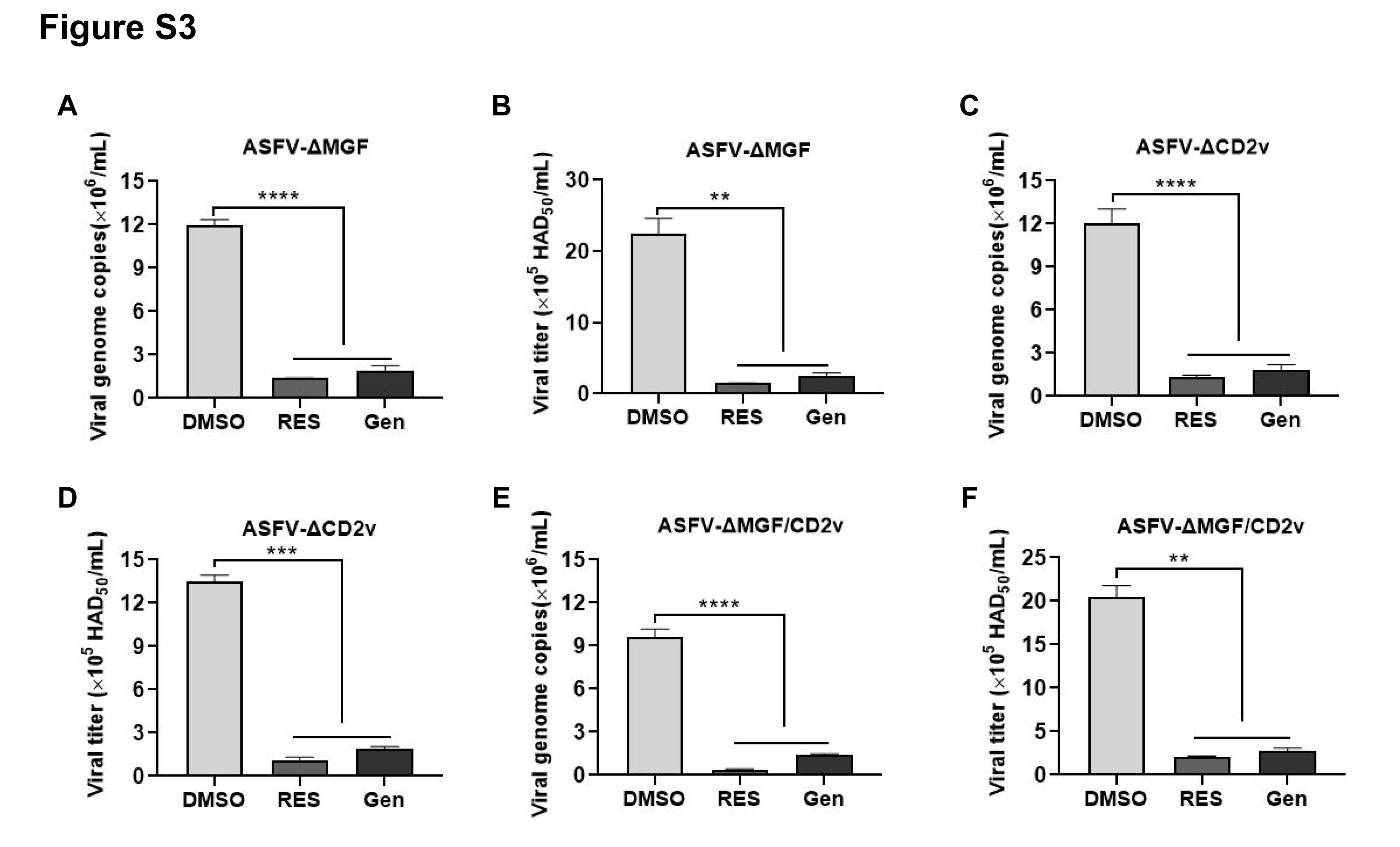

Supplement: Figure S3.tif [file TEMI_A_2469662_SM8776.tif]

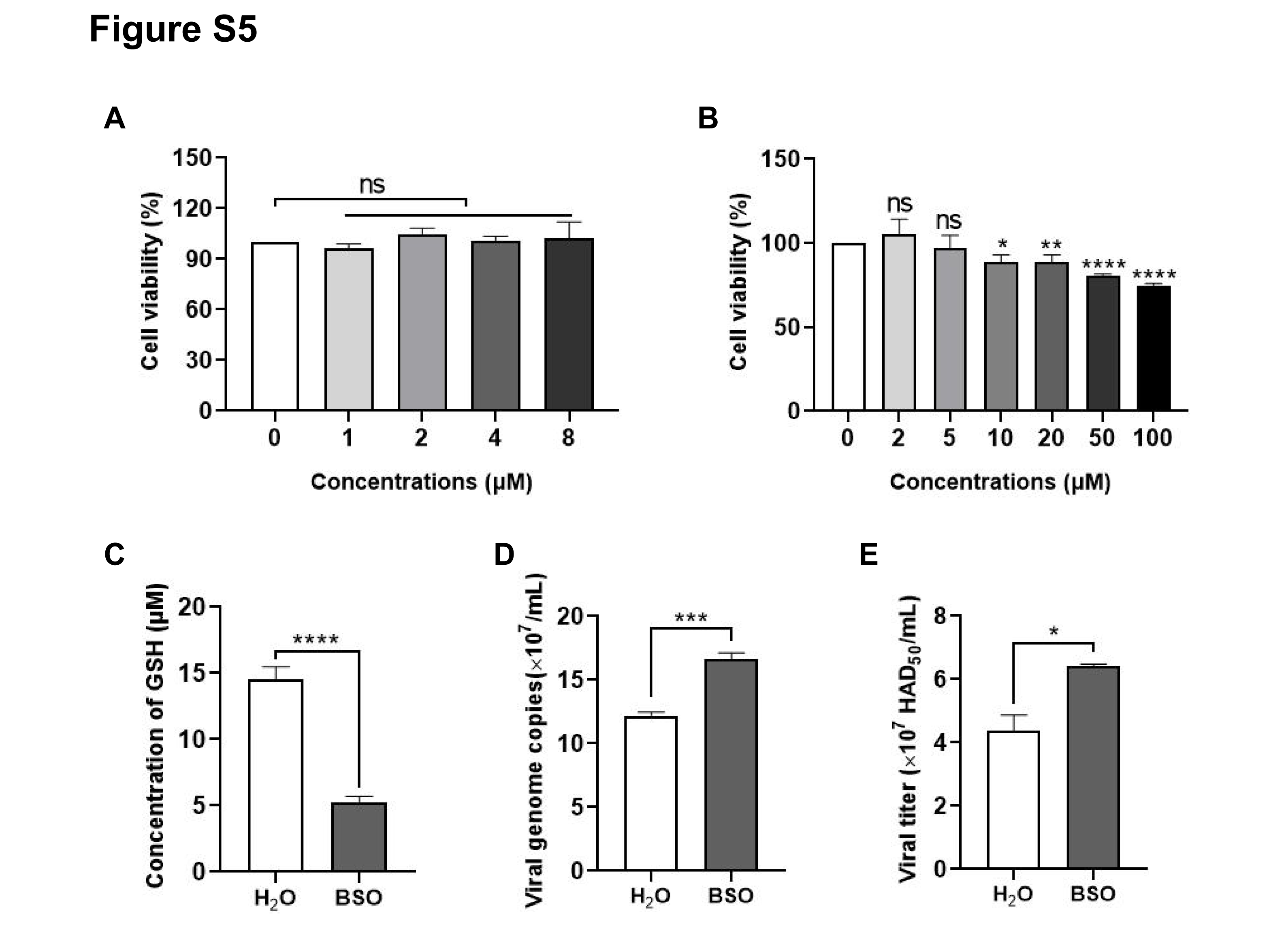

Supplement: Figure S5.tif [file TEMI_A_2469662_SM8774.tif]

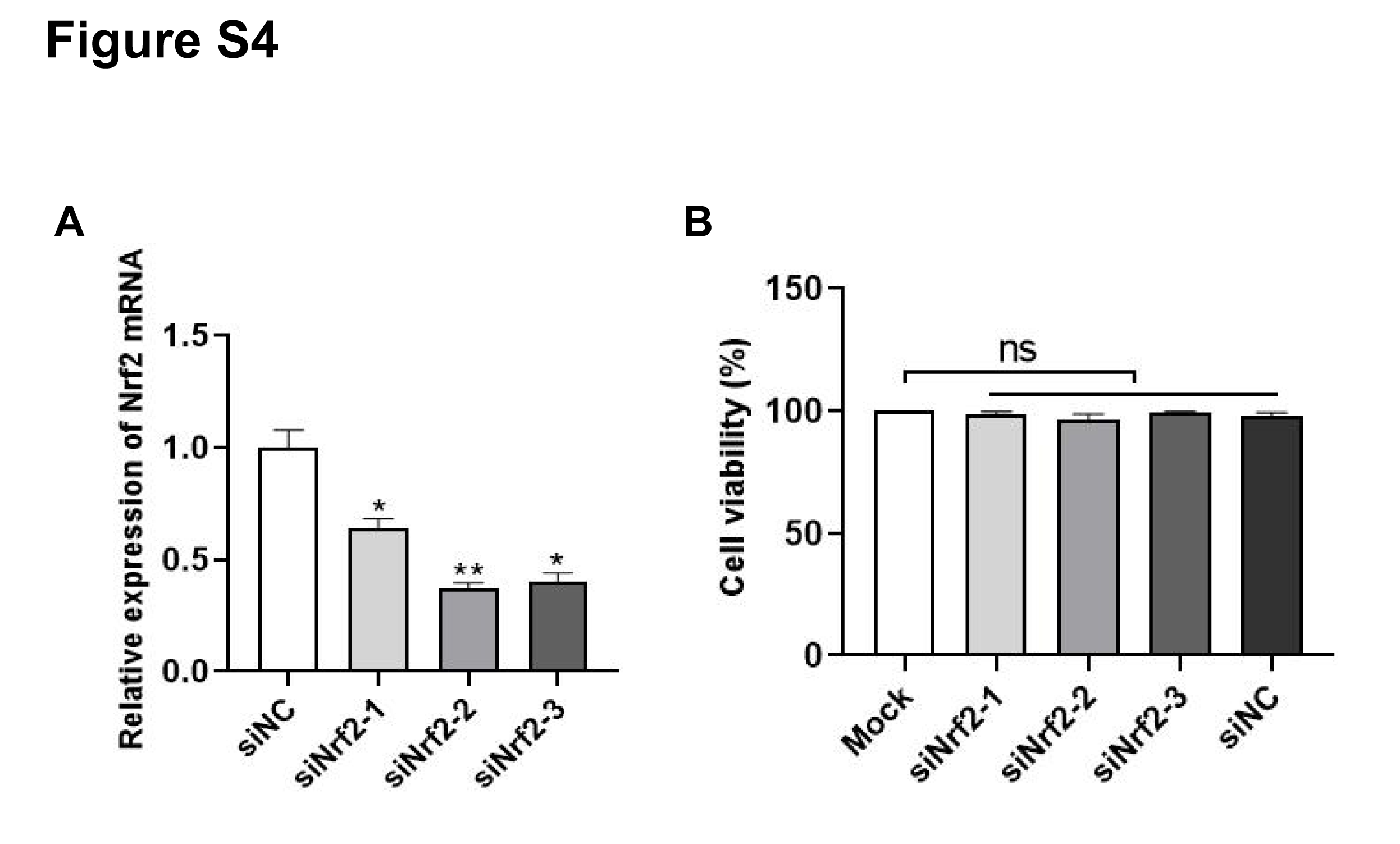

Supplement: Figure S4.tif [file TEMI_A_2469662_SM8772.tif]
