# Supplementary material for: Resveratrol inhibits African swine fever virus replication via the Nrf2-mediated reduced glutathione and antioxidative activities
Source: Emerg Microbes Infect. 2025 Feb 18;14(1):2469662. doi: 10.1080/22221751.2025.2469662 (PMC11878180; doi:10.1080/22221751.2025.2469662)
Supplement: Table S1.docx [file TEMI_A_2469662_SM8775.docx]

**Table S1 The primers used for RT-qPCR**

| **Primers** | **Sequence（5′-3′）** | **Description** |
| --- | --- | --- |
| pNrf2-F | TCCAGAAACCAAACCGACAG | Used in qPCR for porcine *Nrf2* gene |
| pNrf2-R | CTGGCTTGAGTCTTCTGTGG |  |
| p*γ*-GCS-F | GAAATACAATGACATCGACCTG | Used in qPCR for porcine *γ-GCS* gene |
| p*γ*-GCS-R | GAGGCTTGAATCTCATCGTCTG |  |
| pSLC7A11-F | CCTGTCACTGTTTGGAGCCT | Used in qPCR for porcine *SLC7A11* gene |
| pSLC7A11-R | ACCACAGTTATGCCCACA |  |
| pGSR-F | ACTATGGCTTTCAGAGTTGTG | Used in qPCR for porcine *GSR* gene |
| pGSR-R | GCCGTGGATGATTTCTATGTG |  |
| pGAPDH-F | GAAGGTCGGAGTGAACGGATTT | Used in qPCR for porcine *GAPDH* gene |
| pGAPDH-R | TGGGTGGAATCATACTGGAACA |  |
