# Supplementary material for: Resveratrol inhibits African swine fever virus replication via the Nrf2-mediated reduced glutathione and antioxidative activities
Source: Emerg Microbes Infect. 2025 Feb 18;14(1):2469662. doi: 10.1080/22221751.2025.2469662 (PMC11878180; doi:10.1080/22221751.2025.2469662)
Supplement: Table S2.docx [file TEMI_A_2469662_SM8773.docx]

**Table S2 Three siRNAs specifically targeting the porcine *Nrf2* gene**

| **Primers** | **Forward (5’-3’)** | **Reverse (5’-3’)** |
| --- | --- | --- |
| siNrf2-1 | GCCUAUAAGUCCCGGUCAUTT | AUGACCGGGACUUAUAGGCTT |
| siNrf2-2 | GCCCAUUGAUCUCUCUGAUTT | AUCAGAGAGAUCAAUGGGCTT |
| siNrf2-3 | GGCUACAUUUCAAUCACUUTT | AAGUGAUUGAAAUGUAGCCTT |
